# Identification and genome reconstruction of abundant distinct taxa in microbiomes from one thermophilic and three mesophilic production-scale biogas plants

**DOI:** 10.1186/s13068-016-0565-3

**Published:** 2016-07-26

**Authors:** Yvonne Stolze, Andreas Bremges, Madis Rumming, Christian Henke, Irena Maus, Alfred Pühler, Alexander Sczyrba, Andreas Schlüter

**Affiliations:** 1 Center for Biotechnology, Bielefeld University, 33615 Bielefeld, Germany; 2 Faculty of Technology, Bielefeld University, 33615 Bielefeld, Germany

**Keywords:** Anaerobic digestion, Biogas, Microbial community, Metagenomics, 16S rRNA gene, Genome binning, *Cloacimonetes* (WWE1), *Thermotogae*, *Fusobacteria*, *Spirochaetes*

## Abstract

**Background:**

Biofuel production from conversion of biomass is indispensable in the portfolio of renewable energies. Complex microbial communities are involved in the anaerobic digestion process of plant material, agricultural residual products and food wastes. Analysis of the genetic potential and microbiology of communities degrading biomass to biofuels is considered to be the key to develop process optimisation strategies. Hence, due to the still incomplete taxonomic and functional characterisation of corresponding communities, new and unknown species are of special interest.

**Results:**

Three mesophilic and one thermophilic production-scale biogas plants (BGPs) were taxonomically profiled using high-throughput 16S rRNA gene amplicon sequencing. All BGPs shared a core microbiome with the thermophilic BGP featuring the lowest diversity. However, the phyla *Cloacimonetes* and *Spirochaetes* were unique to BGPs 2 and 3, *Fusobacteria* were only found in BGP3 and members of the phylum *Thermotogae* were present only in the thermophilic BGP4. Taxonomic analyses revealed that these distinctive taxa mostly represent so far unknown species. The only exception is the dominant *Thermotogae* OTU featuring 16S rRNA gene sequence identity to *Defluviitoga tunisiensis* L3, a sequenced and characterised strain. To further investigate the genetic potential of the biogas communities, corresponding metagenomes were sequenced in a deepness of 347.5 Gbp in total. A combined assembly comprised 80.3 % of all reads and resulted in the prediction of 1.59 million genes on assembled contigs. Genome binning yielded genome bins comprising the prevalent distinctive phyla *Cloacimonetes*, *Spirochaetes*, *Fusobacteria* and *Thermotogae*. Comparative genome analyses between the most dominant *Thermotogae* bin and the very closely related *Defluviitogatunisiensis* L3 genome originating from the same BGP revealed high genetic similarity. This finding confirmed applicability and reliability of the binning approach. The four highly covered genome bins of the other three distinct phyla showed low or very low genetic similarities to their closest phylogenetic relatives, and therefore indicated their novelty.

**Conclusions:**

In this study, the 16S rRNA gene sequencing approach and a combined metagenome assembly and binning approach were used for the first time on different production-scale biogas plants and revealed insights into the genetic potential and functional role of so far unknown species.

**Electronic supplementary material:**

The online version of this article (doi:10.1186/s13068-016-0565-3) contains supplementary material, which is available to authorized users.

## Background

The reorientation of the global energy industry towards renewable energy sources is one of the major challenges of this century. The development of techniques using these resources contributes to the reduction of traditional fossil fuel usage [1]. Within the agricultural sector of renewable energy sources, energy generation by decomposition of organic materials has become one of the most important techniques. In particular, the production of biogas represents an economically attractive technology to generate bioenergy [2].

In general, organic material is anaerobically decomposed by complex consortia of microorganisms. The final product of this fermentation process is biogas with methane as the main compound. Several studies investigated and characterised the microbial community composition of agricultural biogas reactors. Among the bacterial community members, those of the classes *Clostridia* and *Bacteroidetes* dominate the biogas microbial subcommunities, followed by *Proteobacteria*, *Bacilli*, *Flavobacteria*, *Spirochaetes* and *Erysipelotrichi*. Within the domain *Archaea*, the methanogenic orders *Methanomicrobiales*, *Methanosarcinales* and *Methanobacteriales* were described to be frequently dominant [3–8].

Usually, the anaerobic fermentation process is practised at mesophilic (35–40 °C) or thermophilic (55–60 °C) conditions. Mesophilic biogas plants are typically fed with energy crops in contrast to thermophilic reactors which also convert complex manure mixtures, industrial food residues and organic household wastes [9]. The digestion process under mesophilic conditions requires less process heat and is described to be stable due to the larger diversity of microorganisms, explaining its broader usage [10–12]. In comparison, thermophilic plants show higher methane content of the biogas, faster process turnover rates and a sanitising effect [10]. Depending on the process conditions, such as temperature and fed substrates, differences in the biogas microbiome have been observed [13, 14].

To analyse the structure and function of biogas communities, high-throughput 16S rRNA gene amplicon as well as metagenome sequencing has been applied frequently [3, 4, 7, 15–18]. Still, the complex microbial consortia involved in anaerobic digestion are not fully understood, since many species of the process are unknown and uncharacterised. Culturing of single species is mostly difficult and does not cover the community’s complexity. For culture-independent functional characterisation of microbial species, genome binning from metagenome sequence data has been done for laboratory scale, but not yet on production-scale biogas fermenters [19].

The aim of this study was to compare community structures of different mesophilic and thermophilic production-scale biogas plants for the identification of distinctive taxa and their functional potential. Taxonomic community profiling was achieved by high-throughput 16S rRNA gene amplicon sequencing, whereas ultra-deep metagenome sequencing, assembly of metagenome reads and subsequent binning of obtained contigs resulted in genome bins providing the basis for genome-centred metabolic reconstructions. Genome bins enabled functional predictions for so far unknown and distinctive taxa of the biogas communities analysed.

## Methods

### Sampling at production-scale biogas plants and DNA extraction

Fermentation samples were taken directly from the main fermenters of four different production-scale biogas plants (BGPs 1, 2, 3 and 4, see Table 1). Before sampling at the sampling devices installed at the BGPs, the reactor content was stirred and dead volumes of the outlet pipelines were discarded. One litre fermentation sludge was then filled into a gas tight bottle, respectively, excess air was removed and the bottle tightly closed with a screw cap. Samples were immediately transferred to the laboratory maintaining the process temperature of the sample and then processed for total community DNA extraction. Whole community DNA was prepared from fermenter samples applying the protocol as follows:

A 26 g aliquot of fermenter sludge was mixed with 50 ml of 1 M phosphate buffered saline solution (PBS, 137 mM NaCl, 2.7 mM KCl, 10 mM Na_2_HPO_4_, 1.8 mM KH_2_PO_4_), centrifuged at 9000×*g* for 5 min, the supernatant discarded. The pellet was resuspended in 50 ml PBS (4 °C), shaken at 400 rpm, centrifuged at 200×*g* for 5 min and the supernatant collected. These steps were repeated three times to wash off the microbial biomass off the substrate fibres. The collected supernatant was centrifuged at 9000×*g* for 5 min, the pellet resuspended in 40 ml PBS and further centrifuged at 5000×*g* for 15 min, supernatant discarded. For cell disruption, the pellet was resuspended in CTAB containing DNA extraction buffer (DEP, described previously in [20], 5 mg Pronase ε (Serva Electrophoresis GmbH, Heidelberg, Germany) and 2 mg RNAse (Qiagen, Germantown, MD, USA) added and shaken at 180 rpm and 37 °C for 1 h. Afterwards, 30 ml of 10 % SDS solution were added and the suspension incubated in a water bath at 65 °C for 2 h, inverting the suspension every 15 min. After centrifugation at 3900×*g* for 10 min, the supernatant was filtered through a folded filter (pore size 15–18 µm) and the filtrate mixed 1:1 (v/v) with a 24:1 chloroform/isoamyl alcohol (v/v) mixture, followed by centrifugation at 8000×*g* and 4 °C for 5 min. The upper phase was taken off, mixed 1:0.7 (v/v) with isopropyl alcohol and left at room temperature for 1 h. The DNA was pelleted by centrifugation at 9000×*g* and 4 °C for 20 min. From here, the NucleoBond AX-G (Macherey–Nagel, Düren, Germany) ion exchanger columns and solutions were used for DNA purification according to the manufacturer’s instructions, starting with resuspension of DNA in 2 ml N2-Buffer and overnight incubation at 60 °C. In the end, the DNA was resuspended in 100 µl TE buffer (1 m mM Tris, 1 mM EDTA, pH 8.0) and overnight incubated at 4 °C. DNA concentration was measured using the NanoDrop 2000 Spectrophotometer (Thermo Scientific, Waltham, USA). The DNA extraction above was done in quadruplicates and two DNA replicates were sent to the DOE Joint Genome Institute (JGI) in Walnut Creek, California, USA for 16S rRNA gene amplicon and metagenomic sequencing. Samples for (chemical) process parameter measurements were taken and analysed separately and independently by the biogas plants’ operators.

### High-throughput 16S rRNA gene amplicon sequencing

To describe and characterise the biogas-producing microbial community composition the high-throughput 16S rRNA gene amplicon sequencing approach was applied as published previously [21]. Library preparation and sequencing were done at the DOE JGI. Briefly, to amplify the hypervariable region V4 of the 16S rRNA gene, the primers 515F 5′-GTGCCAGCMGCCGCGGTAA-3′ and 806R 5′-GGACTACHVGGGTWTCTAAT-3′, covering the domains *Bacteria* and *Archaea* [21], multiplex identifier (MID) tags and Illumina-specific sequencing adaptor sequences were used. Afterwards, the fragments of expected length (approx. 300 bp) were amplified by PCR. Obtained PCR products were purified with AMPureXP^®^ magnetic beads (Beckman Coulter GmbH, Brea, CA, USA). Qualitative and quantitative analysis of the generated 16S rRNA gene amplicons was performed using the Agilent 2100 Bioanalyzer system (Agilent, Santa Clara, CA, USA) and afterwards pooled together in equimolar amount. Finally, the constructed amplicon libraries were sequenced on the Illumina MiSeq system applying the paired-end protocol.

## 16S rRNA gene sequence processing and quality control

The raw 16S rRNA gene sequencing reads were preprocessed by JGI’s iTagger amplicon analysis pipeline as to perform quality control. It was used to remove contaminants, e.g. PhiX control, sequencing library adapter dimers, etc., deplete sequencing primers and merge read pairs. Further contaminants removal was performed by DUK (v1.05) [22]. Read pairs with at least one read matching against the PhiX genome or the Illumina-specific sequencing artefact library were removed from the library. Adapter trimming was performed through cutadapt (v1.2.1) providing the sequences of the sequencing primers 515F and 806R [23]. Finally, Flash (v1.2.6) was used for iterative read pair merging through consecutive trimming of the read pairs until these could be merged by removing failing read pairs from the library and a final filter with a threshold of 0.3 errors per 100 bp [24]. Before subsequent analysis with the QIIME NGS analysis pipeline [25], the eight libraries were initially merged into a sample tagged QIIME accessible format, including an additional quality control step checking for min base quality of 20 on Phred scale and truncating sequences if necessary.

### Operational taxonomic unit (OTU) clustering and taxonomic classification of 16S rRNA gene sequences

Further analysis of the 16S rRNA gene reads was performed within the QIIME analysis pipeline for operational taxonomic unit (OTU) clustering and subsequent taxonomic classification of the OTU representatives. From these OTUs, distinctive taxa per sample have been identified and investigated more closely. The pre-filter +4 step open reference based OTU picking workflow from QIIME v1.9.1 was used in combination with Usearch (v7.0.1090, 64bit) and Greengenes 16S rRNA gene database (v13_08, 97 % identity) as reference dataset [26, 27]. The representative sequences of each OTU were aligned using PyNAST (v0.1) [28], where OTUs with sequences failing to be aligned were removed from the final OTU table. Hence, the community profiles were corrected with CopyRighter (v0.46) [29] to account for different 16S rRNA gene copy numbers within the microbial community. The OTU representatives of the four distinctive phyla were placed into the All-Species Living Tree LTPs123 [30]. Sequences were aligned using the SINA alignment service v.1.2.11 online. The LTPs123 tree and SINA alignments were loaded into ARB [31] and sequences placed into the existing LTP tree using ARB’s parsimony method.

### Preparation and metagenome sequencing of total DNA from biogas-producing microbial communities

Library preparation and sequencing from total DNA were done at the DOE JGI. For sequencing purposes, 100 ng of total DNA was sheared to 270 bp fragments using a focused-ultrasonicator (Covaris, Woburn, MA, USA). The DNA fragments were purified and size selected using SPRI beads (Beckman Coulter, Brea, CA, USA). Obtained fragments were blunt-end-repaired, phosphorylated and A-tailed. Subsequently, T-tailed adapters, containing sequences used during cluster formation and Illumina compatible adapters (IDT, San Jose, CA, USA), were ligated to the purified DNA fragments applying the KAPA-Illumina library creation kit (KAPA Biosystems, Wilmington, MA, USA). The prepared sample libraries were quantified applying the KAPA Biosystem’s next-generation sequencing library qPCR kit (KAPA Biosystems, Wilmington, MA, USA) and run on the Roche LightCycler 480 real-time PCR instrument (Roche Basel, Switzerland). Sequencing of the libraries was performed on the Illumina HiSeq 2000 sequencer using the Illumina TruSeq SBS v3-HS kit, following a 2 × 150 indexed high output run protocol.

### Metagenome assembly and binning

To reconstruct low-abundance community members and to facilitate downstream genome binning, all sequencing data was combined after quality control (JGI QC pipeline: sequencing artefact removal, removal of reads containing ambiguous (N) bases and filtering based on quality). Ray Meta (v2.3.0) [32] was used for assembly of the pooled sequencing data of all samples, using a *k*-mer size of 31. To estimate the inclusivity of our metagenome assembly, all sequencing reads were aligned to the assembled contigs with Bowtie 2 (v2.2.4) [33]. SAMtools (v1.0) [34] was used to convert SAM to BAM, sort the alignment file and calculate read mapping statistics.

The gene prediction tool Prodigal v.2.6.0 [35] was used to predict genes on assembled contigs larger than 1 kb. Predicted protein sequences were compared to NCBI’s database using the BLASTP mode of DIAMOND [36]. The resulting output file was loaded into MEGAN5 [37] for taxonomic classification of each gene sequence.

To divide the metagenome assembly into genome bins, MetaBAT (v0.21.3) [38] was used in its very specific mode. Completeness, contamination, and strain heterogeneity were estimated with CheckM (v1.0.4) [39], using sets of clade-specific single-copy marker genes. Genome bins of distinct taxa were identified by (A) counting the aforementioned taxonomic assignments on gene level, and (B) running taxator-tk (v1.2.1; binning-workflow-fasta-blast.sh) [40] to additionally assign a taxon label on contig level. These two approaches were largely in agreement, identifying high-confident genome bins for the taxa of interest. For each taxon, we considered only the most complete and less contaminated genome bin, as estimated by CheckM, for further analyses.

Genome bins were annotated and analysed within the GenDB 2.0 annotation system [41], additionally using the KEGG pathway mapping and BLASTP tool implemented in the system. For gene content comparisons, genome bins and corresponding reference genomes were analysed using the EDGAR 2.0 software [42]. Here, orthologous genes (hereafter referred to as ‘shared genes’) that the compared genomes have in common and singletons (hereafter referred to as ‘unique genes’) that do not have any orthologous counterpart in the respective reference genome were determined based on BLASTP.

## Results and discussion

### Parameters of the three mesophilic and one thermophilic industrial biogas plants analysed

In this study, four different production-scale biogas plants (BGPs) were compared on taxonomic and functional level, based on high-throughput 16S rDNA amplicon and ultra-deep metagenome sequencing. The BGPs analysed are located in North-Rhine-Westphalia, Germany, and regarding their construction, mainly differ in the number of fermenters, size, process temperature and fed substrates. The main fermenters of the biogas plants BGP1, BGP2 and BGP3 are continuously stirred tank reactors (CSTRs), while in the thermophilic BGP4 mixing of the substrate is achieved by pumping it through the reactor. Regarding the operating temperature, BGP4 is a thermophilic biogas plant (54 °C), whereas the other three BGPs were operated under mesophilic conditions (approx. 40 °C). The substrates of all four biogas plants were based on maize silage with the addition of different manure types. In BGP2 and 4, grass silage was also added and BPG1 is unique due to its fermentation of sugar beet as substrate. All BGPs showed stable biogas production and process parameters around the time of sampling (data not shown).

To interpret microbial compositions within the fermenters, other physico-chemical parameters are of importance. Table 1 summarises all parameters of the four fermenters, measured around the time of sampling, and corresponding optimal parameter ranges as taken from different sources [43–45]. Almost all measured parameters of the four BGPs are in the recommended optimal range. One exception is the total inorganic carbon (TIC) value of BGP2, which is above the recommended level and also the volatile organic acids (VOA) concentration is relatively high, resulting in a VOA/TIC ratio that is within the range of 0.11 to 0.6. This indicates that the system is well buffered [46]. Other characteristics are the acetic acid equivalents (HAC-eq) of BGP2 and 4, which are below the optimal range. However, all other parameters including ammonium/ammonia concentrations of these BGPs are within the optimal range. Only BGP2 and 3 have ammonium/ammonia concentrations in the higher range of the optimum and are considered here as ammonia-stressed. BGP4 shows the highest biogas per kg of organic dry matter (l/kg oDM) output and also shows the highest percentage of methane. Both findings are in accordance with increased methane content of thermophilic biogas plants found in the literature [10, 11, 47] indicating that thermophilic biogas plants generally have higher biogas outputs, due to heat-induced increase of enzymatic activity. This is also supported by the data in this study. The finding that the other biogas outputs are only slightly lower, especially for BGP2, shows that also other process parameters, such as substrate type and pH, have a significant influence on the microbial community and the biogas output, which would be consistent with the literature [10, 12, 48, 49].

**Table 1:** Physico-chemical characteristics and fed substrates of the four different biogas plants analysed in this study and optimal ranges of some of the parameters

Parameters	Optimal range^b^	BGP1	BGP2	BGP3	BGP4
pH	6.8–8.0	7.7	7.8	7.53	7.8
VOA (mg/l)	2050–6500	4876	5093	3391	3300
TIC (mgCaCO_3_/l)	8500–15,000	11,040	15,928	14,714	11,600
VOA/TIC	0.11–0.6	0.45	0.32	0.23	0.28
NH4-N (g/kg)	1.2–4.0	1.9	2.32	3.15	n.d.
HAC-eq (g HAceq/l)^a^	1.3–1.9	2.03	0.40	n.d.	0.57
Temperature (°C)	n.d.	40 (mesophilic)	40 (mesophilic)	40 (mesophilic)	54 (thermophilic)
Fed substrates (%)	n.d.	Maize silage (45), sugar beet (22), poultry manure (33)	Maize silage (50), grass (10), poultry/pig/cattle manure (40)	Maize silage (67), pig manure (33)	Maize silage (60), grass (30), pig manure (10)
Retention time (days)	n.d.	92	74	81	28
Biogas yield (l/kg oDM)	n.d.	609.87	644.5	528.5	658.11
% Methane	n.d.	49.60	52.24	52.4	56

*VOA* Volatile organic acids; *TIC* Total inorganic carbon; *oDM* Organic dry matter; *n.d.* No data

^a^Acetic acid equivalent calculated from fermentation acids acetic acid, propionic acid, butyric acid, iso-butyric acid, valeric acid, isovaleric acid, caproic acid

^b^Optimal range of parameters based on [43–45]

### Sequencing results for 16S rRNA gene amplicons and metagenomes

To study the taxonomic microbial community compositions of the four studied BGPs, high-throughput 16S rRNA gene sequencing was done in duplicates on total community DNA extracted from reactor samples of each BGP. The microbial taxonomic composition based on the 16S rRNA gene sequencing data was determined using the QIIME software package and additional CopyRighter analysis for gene copy number corrections. 16S rRNA gene amplicon sequencing and quality control (QC) results are summarised in Table 2. To determine the functional potential of the communities, metagenome sequencing was done, of which statistics and QC results are shown in Table 3. In total, approx. 2.3 billion reads (347 Gb; Table 3) were generated, the deepest sequencing of biogas community metagenomes so far.

**Table 2:** 16S rRNA gene amplicon sequencing and quality control (QC) results

Sample	Replicate	No. of read pairs	No. of bases	No. of QC read pairs	No. of QC QIIME read pairs	% QC read pairs
BGP1	1	94,167	47,270,830	92,820	91,902	97.59
	2	96,103	48,243,706	94,970	94,222	98.04
BGP2	1	203,848	102,331,696	200,788	199,138	97.69
	2	84,099	42,217,698	83,077	82,448	98.04
BGP3	1	81,394	40,859,788	80,398	79,681	97.9
	2	93,699	47,036,898	91,986	91,078	97.2
BGP4	1	84,704	42,521,408	83,366	82,623	97.54
	2	90,079	45,219,658	88,592	87,797	97.47
Total	–	828,093	415,701,682	815,997	808,889	97.68

**Table 3:** Metagenome sequencing and quality control (QC) results and sequence read archive (SRA) accession numbers

Sample	Replicate	No. of raw reads	No. of raw bases	No. of QC’ed reads	No. of QC’ed bases	SRA accession
BGP1	1	267,749,142	40,162,371,300	256,033,246	38,404,986,900	SRA357211
	2	289,930,844	43,489,626,600	276,028,796	41,404,319,400	SRA357213
BGP2	1	298,185,500	44,727,825,000	283,504,064	42,525,609,600	SRA357208
	2	281,693,590	42,254,038,500	277,123,112	41,568,466,800	SRA357209
BGP3	1	242,121,112	36,318,166,800	208,532,304	31,279,845,600	SRA357214
	2	338,184,952	50,727,742,800	326,116,028	48,917,404,200	SRA357221
BGP4	1	307,971,670	46,195,750,500	288,040,900	43,206,135,000	SRA357222
	2	290,604,188	43,590,628,200	271,494,384	40,724,157,600	SRA357223
Total	–	2,316,440,998	347,466,149,700	2,186,872,834	328,030,925,100	_

### The microbiomes of one thermophilic and three mesophilic production-scale biogas plants determined by high-throughput 16S rRNA gene amplicon sequencing

#### Similarities and differences of the microbiomes prevailing in the four analysed BGPs

**Fig. 1:**
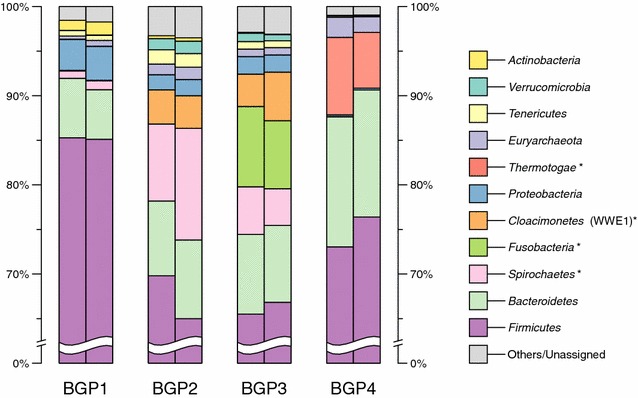
Taxonomic profiles of the four biogas plants (BGPs) on phylum level, based on 16S rRNA gene amplicon sequencing. The respective relative abundances of the replicates for each BGP are shown. Four taxa, distinctly and abundantly present in one or two of the BGPs, were identified: *Thermotogae, Fusobacteria*, *Spirochaetes* and *Cloacimonetes*, highlighted with *asterisks*

To analyse and interpret the taxonomic structure of the microbial communities residing in the three mesophilic and one thermophilic biogas plants, OTU clustering of the 16S rRNA gene sequence data was done for two biological replicates of each BGP. OTUs were clustered on taxonomic ranks from phylum to genus level, calculating their respective percentage share within the respective sample. Figure 1 shows the microbial taxonomic profile for each replicate, with percentage shares for each phylum. The vast majority of taxa prevailing in all four BGPs was assigned to the bacterial superkingdom with between 97.37 % in biogas plant 4 (replicate 1) and 99.19 % (replicate 2), while *Archaea* have a share of between 0.36 % (BGP1) and 2.25 % (BGP4). These results are in accordance with other 16S rRNA gene sequencing studies addressing microbial communities of anaerobic methane-producing reactor systems [8, 50, 51]. Compared with BGP1, 2 and 3, BGP4 shows the lowest diversity among bacterial and archaeal taxa based on the Shannon index of 7.5, 7.4, 6.8 and 5.7, respectively. This is most likely due to the higher process temperature of BGP4. For similar thermophilic systems, it has been shown that the temperature has the main influence on microbial community structures [52–54]. Regarding temperature differences between the three mesophilic and the only thermophilic BGPs, community profiles in general reflect previous findings obtained for similar anaerobic reactor systems [50, 55, 56].

Within the bacterial superkingdom, ten phyla were identified in all BGPs, namely *Firmicutes*, *Spirochaetes*, *Bacteroidetes*, *Proteobacteria*, *Tenericutes*, *Actinobacteria*, *Synergistetes*, *Fibrobacteres*, *Chloroflexi* and OP9 division (Fig. 1). The proportions of these phyla differ considerably between the four BGPs. Sequences assigned to the phylum *Firmicutes* are less abundant in BGP2 and 3 in comparison to BGP1 and 4, with BGP1 having the highest share (Fig. 1). Their dominance in biogas and other fermentation reactors with cellulose-rich substrates was found frequently, underlining their importance and specific adaptation abilities [3, 5, 56–58]. In BGPs 1, 3 and 4 members of the phylum *Bacteroidetes* are the second most abundant bacterial group, while in BGP2 they feature a lower relative abundance, due to a slightly higher proportion of *Spirochaetes*. The relatively high abundance of *Bacteroidetes* in BGP4 is surprising, as it has been shown that members of this phylum are sensitive to high temperatures [57, 59, 60].

The archaeal superkingdom in all samples exclusively comprises the phylum *Euryarchaeota* (Fig. 1) that on class level, is represented by *Methanomicrobia* (0.31–1.84 %), *Methanobacteria* (0.01–0.34 %) and *Thermoplasmata* (0.03–0.08 %) (data not shown). In all four BGPs, the order *Methanomicrobiales* is the most abundant, with *Methanoculleus* being the dominant genus accounting for 0.3 % (BGP1) to 1.8 % (BGP4) of all assigned sequences (not shown). This genus was found dominant in several other mesophilic and thermophilic biogas-producing communities and may outcompete other *Archaea* due to a broad temperature optimum spectrum (20–55 °C) and higher growth rate [4, 8, 18, 46, 50, 51, 61, 62]. However, the genus *Methanothermobacter* (order *Methanobacteriales*) is present only in the thermophilic BGP4, with a share of 0.29 % (not shown). Members of this genus are known to be thermophilic and often are dominant in thermophilic methane-producing microbial consortia [52, 60, 61]. In general, the mostly hydrogenotrophic genera dominated methanogenic communities indicate that they are mostly based on CO_2_ and formate as electron acceptors and H_2_ as electron donors for methanogenesis in all four BGPs. High affinities towards hydrogen and a better adaptation to lower hydrogen pressures may explain the strict dominance of the hydrogenotrophic metabolism [61, 62]. It is also possible that aceticlastic methanogens have been inhibited by elevated ammonium/ammonia concentrations in the reactors BGP2 and 3 as they all are in the upper part of the optimal range regarding this process parameter (Table 1). In case of the thermophilic BGP4, the operating temperature also drives methanogenesis towards the hydrogenotrophic mode, as it is thermodynamically more favourable and it has also been reported that hydrogenotrophic methanogenesis is the dominant pathway in thermophilic methane-producing reactors [45, 51, 54, 59, 60].

When comparing the phyla percentages of the four BGPs, it is noticeable that members of the *Thermotogae* are present in only one, *Fusobacteria* and *Cloacimonetes* members in only two and *Spirochaetes* members are present in only three of four BGPs, the latter being highly abundant in only two BGPs (Fig. 1). To further investigate these distinct taxonomic features, community profiles were followed down towards deeper taxonomic levels.

#### The phylum *Thermotogae* and its members are present only in the thermophilic BGP4

The taxonomic profile of BGP4 features a high share of *Thermotogae* (approx. 7.5 %), which are absent (BGP2/3) or present only in very low abundances (approx. 0.02 %, BGP1) in the mesophilic BGPs (Fig. 1). All 16S rRNA gene sequences assigned to the phylum *Thermotogae* were classified as class *Thermotogae*, order *Thermotogales* and family *Thermotogaceae* (see Add. File 1).

Members of the phylum *Thermotogae* were also found to be present in similar reactors operated under mesophilic conditions (31–41 °C) [18, 54, 63, 64], but were identified more frequently and in higher abundances in anaerobic reactors operated at thermophilic temperatures (50–60 °C) [54, 59, 64]. The results of this study reflect previous findings, which can be explained by the temperature optimum for *Thermotogae* members being mostly in the range of approx. 50–60 °C. The existence of mesophilic (‘mesotoga’) and hyperthermophilic members of the phylum were also described and at least for the latter group, several genome sequences are available in the literature [65–68]. However, the prevailing temperature of 54 °C in BGP4 meets the preferred temperature demands of thermophilic *Thermotogae* species, explaining their presence in this reactor. Moreover, their high share and the lower abundances of other bacterial phyla (i.e. *Proteobacteria*, *Tenericutes, Cloacimonetes*, see Fig. 1) is most likely due to the adaptation-based outcompeting effect at the expense of those community members that are not adapted to high temperatures. In anaerobic fermentation of biomass, *Thermotogae* members are involved in the degradation of cellulose and highly complex polysaccharides, producing acetate, carbon dioxide and hydrogen, and therefore are involved in hydrolysis and acetogenesis [64, 67, 69]. Additionally, members of the phylum *Thermotogae* are thought to be syntrophically associated with methanogenic *Archaea*, and therefore are essential for the maintenance of methane production [65, 69, 70]. It can be assumed that *Thermotogae* species within BGP4 have a similar metabolism and syntrophic character compared to reference species.

#### The phylum *Fusobacteria* and its members are solely present in the mesophilic BGP3

The taxonomic profile of BGP3 is distinct due to the presence of *Fusobacteria,* having an average share of 8.3 % of all rRNA gene sequences in the replicates, compared to 0–0.02 % in the communities of the other BGPs (Fig. 1). All *Fusobacteria* sequences from BGP3 were assigned to the class *Fusobacteria* and the order *Fusobacteriales* (see Additional file 1).

*Fusobacteria* were also found in other studies focusing on microbial communities in biogas-producing reactors, but with a share below 2 % [6, 18]. Naturally, these anaerobic, mesophilic bacteria are found, e.g. in the mouth and gastrointestinal (GI) tract of humans, rats, cattle, sheep and chicken, were they can cause severe diseases [71–73]. Therefore, it can be assumed that *Fusobacteria* in BGP3 originated from the fed cattle manure. Frequently, the *Fusobacterium* species *F. necrophorum* and *F. nucleatum* are associated with infections in humans and animals [71], but neither of these species was identified within BGP3.

Since the focus of research is more on the pathogenic *Fusobacteria* species, nothing is known about the role of *Fusobacteria* within methane-producing biogas plants. Hence, the lack of reference genomes of species playing a role in these systems hampered a taxonomic classification on lower ranks. Their absence in BGP4 can be explained by the high temperature, since these bacteria most likely are adapted to the mesophilic body temperatures of their hosts. One explanation for their absence in BGPs 1 and 2, despite added manure, could be the higher pH values of corresponding fermentation samples. It is known that *Fusobacteria* prevailing in the microbial communities of human oral biofilms live at fluctuating pH values of 6.3–7.0 [74]. BGP3 had the lowest pH value (7.53) of all BGPs analysed. However, due to the lack of information on the phylum *Fusobacteria* in the context of biomass fermentation, it cannot be understood clearly why these bacteria are present only in BGP3.

#### The phylum *Spirochaetes* and its members are most abundant only in the mesophilic plants BGP2 and BGP3

The biogas plants 2 and 3 both have the highest proportion of *Spirochaetes*, with average shares of 10.6 and 4.7 % of all 16S rRNA gene sequence reads, respectively, while in BGP1 the average share of *Spirochaetes* is 0.92 % and BGP4 almost completely lacks this phylum (0.01 %) (Fig. 1). All *Spirochaetes* sequences from BGP3 and the majority in BGP2 were assigned to the class *Spirochaetes* and the order *Spirochaetales* (see Additional file 1).

Not much is known about non-pathogenic *Spirochaetes* since most members of this phylum are described to be human or animal pathogens [75]. Non-pathogenic *Spirochaetes*, especially *Treponema* species, can be found in termite guts, where they form a symbiosis with their hosts [76, 77]. In the context of anaerobic digestion of municipal and/or agricultural wastes, representatives of this phylum were mostly found in mesophilic anaerobic reactors fed with swine manure and cellulose-rich substrates. It is assumed that within these environments *Spirochaetes* are involved in cellulose degradation [56, 78, 79]. In BGP2 and 3, swine manure and the cellulose-rich substrates maize and grass silage were fed, which may explain the high abundance of *Spirochaetes* in the respective microbial communities. Although BGP4 is fed with pig manure, the phylum *Spirochaetes* is underrepresented which may be explained by the thermophilic conditions prevailing in BGP4. *Spirochaetes* are probably not adapted to higher temperatures, and therefore, their function was adopted by other community members such as for example *Thermotogae* species.

#### The candidate phylum *Cloacimonetes* (WWE1) was only identified in the mesophilic biogas plants BGP2 and BGP3

In the taxonomic profiles of the BGPs 2 and 3, an average share of 3.7 and 4.5 % of all 16S rRNA gene sequences was assigned to the candidate phylum *Cloacimonetes* (WWE1), respectively, while in the BGP1 and 4, this phylum is almost absent with an average of max. 0.05 % (Fig. 1). All *Cloacimonetes* (WWE1) sequences from BGP3 and the majority in BGP2 were assigned to the class *Cloacamonae* and the order *Cloacamonales* (see Additional file 1). Since there is very little information about the phylum *Cloacimonetes* and its lower taxonomic levels, there are no alternatives to this class and order that were proposed by the RDP classifier used to classify the 16S rRNA gene sequence data.

The phylum was identified in 2005, named WWE1 (for waste water for Evry 1) and later renamed candidate phylum *Cloacimonetes* (WWE1) [80, 81]. Recently, it has been characterised as a separate phylum, belonging to the *Fibrobacteres*-*Chlorobi*-*Bacteroidetes* (FCB) superphylum and proposed to be a sister group of the phylum *Spirochaetes* [81]. Due to its novelty, missing reference genomes most probably hampered a deeper taxonomic classification of *Cloacimonetes* (WWE1) species residing in BGPs 2 and 3. Members of this phylum are mostly present in anaerobic habitats, such as biogas plants and the porcine digestive tract. It is assumed, and evidence is increasing, that corresponding bacteria are involved in mostly cellulose or sugar degradation, directly derived from cellulose and produce primarily acetate and hydrogen, which are further metabolised by their syntrophic partners, methanogenic *Archaea* [3, 78–84].

No reports exist for the presence of candidate phylum *Cloacimonetes* (WWE1) members in thermophilic environments. This supports the assumption that the temperatures in BGP4 exceed the optimum for species belonging to this candidate phylum. In BGPs 2 and 3, a lower share of *Firmicutes*, also being involved in hydrolysis, compared to the other two BGPs, can be observed. Possibly, *Cloacimonetes* (WWE1) species partly complemented the function of *Firmicutes* species. Recently, *Cloacimonetes* species were shown to increase in their abundance when the ammonium/ammonia concentration was high. This indicates that they can adapt to this condition and even seem to benefit from it when the system is ammonium/ammonia adapted [82]. Regarding the addition of swine manure, this may also explain the presence of *Cloacimonetes* species in BGPs 2 and 3, as these show the highest ammonia concentrations, especially BGP3, whose values are in the higher range of the optimum.

### Most of the dominant distinctive taxa are not represented by closely related reference species in databases

**Fig. 2:**
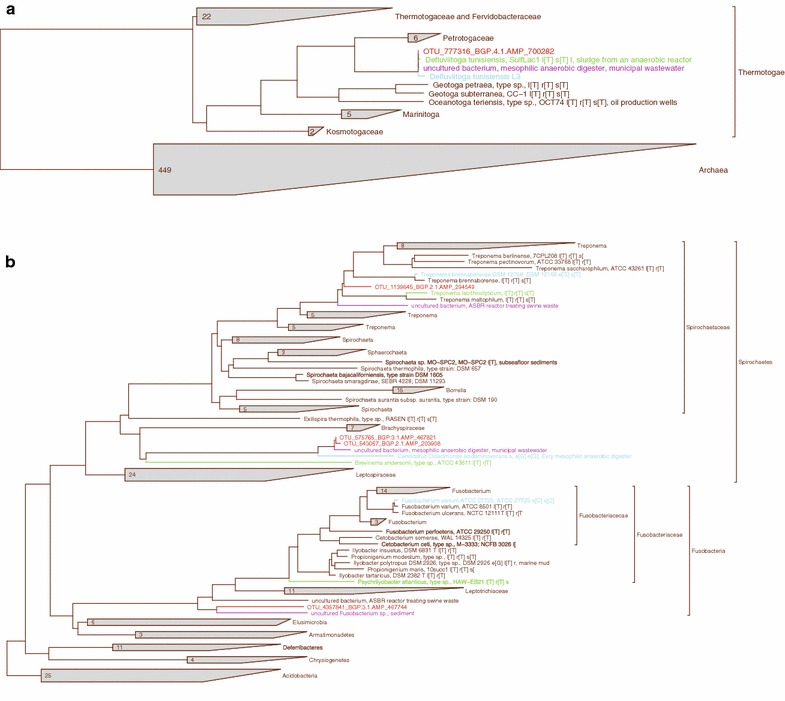
Partial phylogenetic trees of all available type strains with operational taxonomic units (OTUs) of *Thermotogae* (**a**), *Fusobacteria*, *Spirochaetes* and *Cloacimonetes* (**b**) taxa of the studied biogas plants and their closest non-type strain relatives embedded. Type strains are in *black*, OTUs are in *red*, their closest relatives in *purple*, their closest sequenced relatives in *blue* and their closest type strain relatives in *green*. 16S rRNA sequence tree construction was done using the ARB software [31]

Taxonomic profiling of the community structure revealed the presence of distinctive taxa that are present only in one or two of the four BGPs. This observation raises the question concerning the function of these distinctive taxa within the trophic network of the biogas plant’s microbial communities. To determine the closest relatives of distinctive taxa, corresponding dominant OTUs deduced from clustering of 16S rRNA gene sequence data were placed in a phylogenetic tree. Figure 2 shows condensed phylogenetic trees considering only the dominant OTUs of the taxa *Thermotogae* (OTU_ 777316, Fig. 2a) *Spirochaetes* (OTU_1139645), *Fusobacteria* (OTU_4357841) and *Cloacimonetes* (OTU_575765 and OTU_543067) (Fig. 2b). The complete phylogenetic tree can be found in the supplementary material (see Additional file 2).

Of the four distinctive phyla, only the dominant OTU of the phylum *Thermotogae* shows close relatedness to an characterised bacterium (purple), followed by the type strain *Defluviitoga tunisiensis* SulfLac1^T^ (green, [85]), and the recently characterised and sequenced non-type strain *Defluviitoga tunisiensis* L3 (blue, [86]) .

The *Fusobacteria* OTU derived from 16S rRNA gene sequence data of BGP3 was placed within a sister group of the *Leptotrichiaceae*. However, its closest relative is an uncultured bacterium belonging to the genus *Fusobacterium* (purple). Interestingly, the closest type strain relative belongs to the genus *Psychrilyobacter* (green), while the closest characterised and sequenced non-type strain relative is *Fusobacterium varium* ATCC 27725 (blue) (accession number NZ_ACIE00000000.2).

The representative OTU 16S rRNA gene sequence from BGP2 belonging to the phylum *Spirochaetes* was placed within the group of the genus *Treponema* of the family *Spirochaetaceae*. For this OTU, the most closely related sequence belongs to the uncultured type strain *Treponema lecithinolyticum* OMZ 684^T^ (green, [87]) that is not further characterised. When comparing the query OTU to all 16S rRNA gene sequences available in the NCBI database, it appeared that the closest non-type strain relative is the genome sequenced bacterium *Treponema brennaborense* (blue, [88]). This indicates that the dominant OTU represents a new species of the genus *Treponema* that needs further characterisation.

The two most dominant representative OTU 16S rRNA gene sequences belonging to the phylum *Cloacimonetes* (WWE1) originate from BGP2 and 3, respectively, and are closely related to each other. They were placed within the phylum *Spirochaetes*, which is inconsistent regarding the newer literature, since *Cloacimonetes* has recently been classified as an autonomous sister group of the *Spirochaetes* [81]. The closest relative of the *Cloacimonetes* OTUs is an uncultured uncharacterised bacterium (purple) and the only characterised and closest relative is *CandidatusCloacamonas acidaminovorans* (blue) [89].

In summary, with the exception of the *Thermotogae* OTU, no clear classification of the dominant OTUs representing the distinctive taxa could be achieved. The lack of suitable reference genomes hampers the classification of these OTUs and accordingly, information on the functional role of these taxa within the biogas process is currently not available. These issues cannot be solved by 16S rRNA gene sequence comparison. Exploration of the corresponding metagenome sequence datasets of the four biogas plants is needed to uncover the functional role of the identified distinctive taxa. This can be achieved by binning of metagenome contigs representing dominant species of the four distinctive taxa, their annotation and comparative analyses using sequenced and characterised relatives (blue, Fig. 2) identified in this section.

### Reconstruction of genomes representing distinctive taxa applying metagenome assemblies and binning

#### Metagenome assembly and binning results

A total of 1.49 Gbp metagenomic data were assembled (Table 4) and contigs were sorted into 532 genome bins, five of which belong to the taxa of interest and met the stringent quality requirements as defined in the “Methods” section: One *Thermotogae* genome bin (206_*Thermotogae*), one *Fusobacteria* bin (175_*Fusobacteria*), one *Spirochaetes* bin (128_*Spirochaetes*) and two *Cloacimonetes* (WWE1) bins (120_*Cloacimonetes*; 244_*Cloacimonetes*) were chosen. The estimated amount of contamination is largely negligible and mostly due to strain heterogeneity (Table 5).

**Table 4:** Assembly and mapping results (contigs >1 kbp)

Total Bases	No. of contigs	N50	Largest contig	No. of genes	% reads of BGP1	% reads of BGP2	% reads of BGP3	% reads of BGP4
1,488,298,777	330,955	10,556	668,635	1,591,820	74.83 75.14	78.07 78.34	81.11 81.29	86.53 86.50

**Table 5:** Genome binning results and statistics

Bin ID	Total bases	% G/C content	No. of contigs	N50	Largest contig	% completeness	% contamination	% strain heterogeneity
206_*Thermotogae*	1,904,666	30.7	277	8541	50,211	82.81	7.37	87.50
175_*Fusobacteria*	2,063,893	26.2	143	26,189	112,070	94.38	3.37	100.00
138_*Spirochaetes*	2,196,644	59.0	86	38,653	114,681	96.48	4.16	100.00
244_*Cloacimonetes*	1,745,914	54.6	101	25,062	99,397	96.70	2.33	75.00
120_*Cloacimonetes*	2,265,914	51.4	162	18,253	44,371	95.60	28.42	97.44

To deduce the metabolism of all five genome bins, they were annotated and analysed in the annotation platform GenDB 2.0 [41]. Encoded enzymes were mapped on KEGG pathways within GenDB to enable metabolic pathway reconstructions. An example of this analysis is given in Additional file 3, showing the coverage of the ‘Alanine, Aspartate and Glutamate metabolism’ KEGG pathway by enzymes encoded in the genome bin assigned to the phylum *Fusobacteria*.

#### The genome bin representing a *Thermotogae* species from the thermophilic biogas plant 4

Analyses of 16S rRNA gene sequences revealed a close relatedness of the dominant *Thermotogae* OTU from the thermophilic BGP4 to the strain *D. tunisiensis* L3 (Fig. 2a). Compilation of a *Thermotogae* genome bin derived from metagenomic contigs of BGP4 and comparison with the *D. tunisiensis* L3 genome was conducted to further determine the degree of similarity and evaluate the binning approach itself. The yielded genome bin assigned to the class *Thermotogae* (Table 5) is covered by 4.45 % of all BGP4 metagenome reads. In addition to the annotation and functional interpretation in GenDB, the genome bin was examined for the presence of genes encoding proteins involved in energy generation of *D. tunisiensis* [90]. Enzymes encoded in the *Thermotogae* bin and predicted to be involved in sugar utilisation and fermentation metabolism are listed in Additional file 4. These analyses indicated that the *Thermotogae* species represented by the bin is able to utilise xylose, glucose, mannose, galactose, lactose, maltose, fructose, ribose and l-lactate with acetate, CO_2_ and H_2_ as end products of the fermentation.

**Fig. 3:**
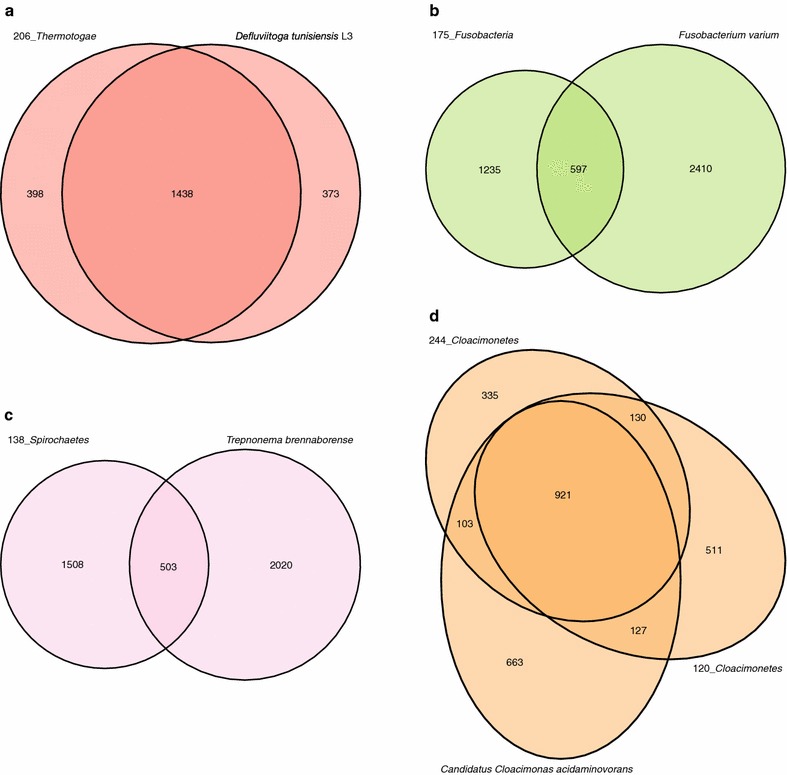
Venn diagrams showing the number of unique and shared genes between the five genome bins and their respective reference strains. Diagrams are shown for genome bins assigned to the phyla *Thermotogae* (206_*Thermotogae*, **a**), *Fusobacteria* (175_*Fusobacteria*, **b**), *Spirochaetes* (138_*Spirochaetes*, **c**) and *Cloacimonetes* (120_*Cloacimonetes*, 244_*Cloacimonetes*; **d**). Venn diagrams were redrawn manually, based on the original EDGAR output, with their areas drawn to scale

Results of the comparative analysis of the annotated *Thermotogae* genome bin and the reference sequence of *D. tunisiensis* L3 using EDGAR were visualized in a Venn diagram (see Fig. 3a, 206_*Thermotogae*). The vast majority of their genes are shared corroborating that the *D. tunisiensis* and the bin genome are closely related. Deeper analysis of the core gene set indicated that both species are anaerobic bacteria featuring a metabolism based on sugar fermentation (see Additional file 4). However, the unique gene set of *D. tunisiensis* L3 indicates that it can utilise a broader spectrum of carbohydrates, since it encodes genes for the import and fermentation of sugars that are missing in the genome bin. Analyses on the *Thermotogae* genome bin’s unique gene set showed that the vast majority (approx. 60 %) could not be functionally classified and the remaining ones do not provide any further information on this strain’s metabolism.

All above mentioned analyses indicate that the species represented by the bin has a metabolism based on sugar fermentation, with acetate, CO_2_ and H_2_ as end products of this process, which has also been predicted for the reference strain *D. tunisiensis* L3. It therefore can be assumed that *Defluviitoga* strains contribute to acetogenesis of biomass digestion within the biogas fermenter. Furthermore, obtained comparison results also support the idea that the analysed *Defluviitoga* species may be syntrophically associated with methanogenic *Archaea* that can utilise CO_2_ and H_2_ for methanogenesis [65, 69, 70]. Additionally, comparative analyses between the genome bin und the highly related reference genome to evaluate the binning approach were done with the outcome that the approach proved valuable and reliable.

#### The genome bin representing a *Fusobacteria* species from the mesophilic biogas plant 3

To analyse the most dominant *Fusobacteria* species of BGP3 on the genomic level, genome bins assigned to corresponding taxa were extracted. The most dominant *Fusobacteria* bin featuring the highest completeness and lowest contamination (Table 5) is covered by 1.4 % of all BGP3 metagenome reads. Since the representative *Fusobacterium* OTU (Fig. 2b) is moderately related to the reference strain *Fusobacterium varium* (see Fig. 2b, blue), the corresponding genome bin was compared to this sequenced and annotated reference genome (NZ_ACIE00000000.2).

The *Fusobacteria* genome bin was annotated, analysed and compared with the *F. varium* genome in GenDB. Results of these analyses suggest that the *Fusobacteria* species represented by the genome bin is an acidogenic bacterium, whose metabolism is mainly based on amino acids as energy and carbon source. This is also known for most *Fusobacteria* species, including the reference strain *F. varium* [91–94], suggesting that they take part in acidogenesis having a metabolism based on sugars and amino acids. Genome bin analysis also indicates that corresponding pathways lead to the production of the end products CO_2_, NH_3_, H_2_, acetate and lactate that can be partly further metabolised by methanogenic *Archaea*. Results of the comparative analysis of the annotated *Fusobacteria* genome bin and the reference sequence of *F. varium* using EDGAR were visualized in a Venn diagram (see Fig. 3b, 175_*Fusobacteria*). It shows that they share a comparatively low number of genes. Further analyses on the unique genes of the *Fusobacteria* bin did not result in additional metabolic information, as for most of them no functional prediction could be obtained (76.03 %). This suggests that the genome bin strain is highly different from other known *Fusobacteria* species and specific genome features remain to be determined.

In conclusion, the analysed *Fusobacteria* species represented by the genome bin most likely is involved in amino acid fermentation and produces CO_2_, NH_3_, H_2_, acetate and lactate, making it an acidogenic bacterium. It may be syntrophically associated with hydrogenotrophic methanogens. Due to a high number of unique unknown genes, this new and uncharacterised species requires further analyses.

#### The genome bin representing a *Spirochaetes* species from the mesophilic biogas plant 3

To analyse the most dominant *Spirochaetes* species of BGP3 on the genomic level, genome bins assigned to corresponding taxa were extracted. The largest and most complete *Spirochaetes* (Table 5) genome bin is covered with 0.2 % of all BGP3 metagenome reads and was chosen for further analyses. Since the representative *Spirochaetes* OTU (Fig. 2b) is moderately related to the reference strain *T. brennaborense* (see Fig. 2b, blue), the corresponding genome bin was compared to this sequenced and annotated reference genome [NC_015500].

Genome annotation and metabolic reconstruction in GenDB revealed that the corresponding *Spirochaetes* species is a hydrolytic bacterium. The presence of a large number of transporters and a number of genes encoding enzymes involved in sugar utilisation indicates that it primarily uses sugars for energy generation, namely glucose, mannose, fructose, rhamnose, xylose, melibiose, stachyose, raffinose and additionally L-lactate. End products of this fermentation were predicted to be acetate, CO_2_ and hydrogen, which can be directly used by methanogenic *Archaea*. Since some *Treponema* species are found in termite guts producing acetate from CO_2_ and H_2_, the presence of the gene (*fhs*) for this reaction’s key enzyme, formyl tetrahydrofolate synthetase [76], was searched by BLAST analysis implemented in GenDB. Its absence indicated that this species is not able to perform homoacetogenesis. Interestingly, no motility genes were found, although it is known that *Spirochaetes* species possess flagella and are motile [95].

To enable a comparison with the reference strain *T. brennaborense*, the EDGAR software was used, resulting in a Venn diagram (Fig. 3c, 138_*Spirochaetes*). It shows that the genomes share a relatively low number of genes, of which 91.54 % were functionally annotated, most being housekeeping genes and others predicted to be involved in sugar metabolism. In contrast, 47.35 % of the unique bin genes are uncharacterised or hypothetical, while 52.65 % were characterised. Of these, many encode for ABC transporters, sugar transporters and enzymes involved in sugar utilisation, underlining the assumption that the corresponding species is particularly dependent on sugars. This and the comparatively low number of shared genes highly suggest that the genome bin represents a new *Spirochaetes* species.

In conclusion, the analysed *Spirochaetes* species represented by the genome bin most likely ferments sugars and produces acetate, CO_2_ and H_2_. It was predicted to utilise a wide range of carbohydrates.

#### The genome bins representing *Cloacimonetes* species from mesophilic biogas plants 2 and 3

To deduce the functional role of dominant *Cloacimonetes* species from BGP2 and 3, the two genome bins representing species, assigned to this phylum with the highest completeness and lowest contamination, were analysed. The *Cloacimonetes* genome bin 1 and 2 are covered by 0.08 % BGP3 reads and 0.23 % BGP2 reads, respectively. The only sequenced species of the phylum *Cloacimonetes*, *CandidatusCloacamonas acidaminovorans* (Fig. 2b), is larger in size and also has a significantly lower GC-content of 37.9 % [89]. *CandidatusCloacamonas acidaminovorans*, whose genome was reconstructed from metagenomic data, may produce hydrogen, and therefore, most likely is syntrophically associated with hydrogenotrophic methanogens [89].

Gene prediction, annotation and interpretation of genome bin 1 in GenDB showed that it lacks genes encoding enzymes involved in the synthesis of eleven amino acids, namely arginine, cysteine, histidine, isoleucine, leucine, lysine, methionine, phenylalanine, tryptophane, tyrosine and valine. Different from what was proposed for species of the candidate phylum *Cloacimonetes* (WWE1) [78], the analysed bin does not have the genetic potential to degrade cellulose or cellobiose. Genome bin 1 possesses genes encoding enzymes for energy generation from glucose via glycolysis, but in addition to this also has the potential to generate energy by degrading the amino acids proline, alanine, aspartate, glutamate, lysine and asparagine with CO_2_ and H_2_ as products. Additionally, corresponding species may be able to produce energy via proton and sodium pumps in combination with hydrogenases. For gaining additional information, proteins involved in fermentation, and energy metabolism compiled in a corresponding study on the reference strain [89] were compared to those encoded by the genome bins (see Additional file 5). It appeared that bin 1 encodes all listed proteins, suggesting that this species can shortly tolerate small amounts of oxygen, but is adapted to an anaerobic lifestyle. Additionally, this species presumably generates energy by Fe-hydrogenases, with H_2_ as end product. Analyses on *Cloacimonetes* bin 2 showed that it was predicted to feature the same metabolism as compared to *Cloacimonetes* bin 1 regarding amino acid fermentation metabolism yielding CO_2_ and H_2_ as products.

To enable further comparison with the reference strain *CandidatusCloacamonas acidaminovorans* (see Fig. 2b), the EDGAR software was used. To further analyse the degree of genetic similarity between both annotated *Cloacimonetes* genome bins and *CandidatusCloacamonas acidaminovorans*, the number of all shared and unique genes was determined. A resulting Venn diagram (Fig. 3d, 120_*Cloacimonetes*, 244_*Cloacimonetes*) shows that all three genomes share the vast majority of genes. Further analyses on the unique genes of *Cloacimonetes* bin 1 and 2 did not result in additional information, since for only 17 and 20 % of these genes, functional prediction could be obtained. These comparative analyses indicate that corresponding microorganisms share a similar metabolism based on utilisation of certain amino acids. However, specific genome features remain to be determined.

In conclusion, the two analysed *Cloacimonetes* species represented by genome bins are most likely amino acid fermenting, CO_2_ and H_2_ producing anaerobes that might be syntrophically associated with hydrogenotrophic methanogens.

## Conclusions

To understand, evaluate and optimise the production process in biogas fermenters, it is crucial to study their microbial communities with all their members and interactions, which are diverse and highly dependent on different process parameters. In this study, three different mesophilic and one thermophilic production-scale biogas plant (BGP) were comparatively characterised. For taxonomic investigation and comparison, the approach of high-throughput 16S rRNA gene sequencing was used. Results showed that microbial communities of biogas plants are taxonomically complex and the process temperature is an important parameter shaping biogas consortia. Still, a core microbiome seems to be present in all BGPs, including taxa belonging to the phyla *Firmicutes* and *Bacteroidetes* and with lower abundances to the *Euryarchaeota*. These are commonly found in BGPs, especially the former two in those fed with cellulose-rich substrates, as they are responsible for the hydrolysis and acetogenesis/acidogenesis steps of anaerobic digestion, while members of the *Euryarchaeota* are involved in methanogenesis. Differences in taxonomic profiles between the BGPs, most probably, are due to adaptations of particular community members to prevailing process parameters, especially when comparing temperature, as the overall diversity is lower within the thermophilic BGP. However, the identification of four highly distinctive phyla characteristic for one (*Thermotogae, Fusobacteria*) or two (*Cloacimonetes, Spirochaetes*) of the biogas plants was notable and represented the main differences between the BGPs. They showed a high prevalence within their respective reactor environment and seemed to be mostly dominated by only a small number of genera.

To additionally uncover the genetic potential of the four studied BGPs, ultra-deep Illumina HiSeq metagenome sequencing was done. In contrast to read-based approaches on microbial metagenomes in the past, a combined assembly of all metagenomes was done in our study. It resulted in a high number of taxonomically and functionally characterised contigs enabling context-based community analyses. Based on the contigs, a genome binning approach was applied successfully. Comparative analyses of the genome bin representing a dominant species belonging to the *Thermotogae* with its closest relative *Defluviitoga tunisiensis* L3 were done. They showed a high similarity and with this confirmed the applicability and reliability of the binning approach. Further exploitation of genome bin information also enabled evaluation of so far unknown biogas species belonging to the phyla *Fusobacteria*, *Spirochaetes* and *Cloacimonetes*. Insights into their genetic potential and putative roles within the biogas fermentation process were obtained. In the past, this was achieved only by cultivation and subsequent genome sequencing or single cell sequencing, which can be difficult especially for taxonomically diverse communities. The assembly based genome binning approach therefore can be seen as an alternative regarding the identification and genetic evaluation of unknown species circumventing the limitations of the methods mentioned before.

This is the first study to use the genome binning approach on deeply sequenced metagenome data originating from different production-scale biogas plants. The combined assembly based binning strategy enabled the identification of five high quality genome bins, representing dominant but mostly unknown species within the complex biogas microbiome. In combination with the taxonomic evaluation of the community by 16S rRNA gene amplicon sequencing and its relation to prevailing process parameters, it allows deep insights into the members’ functional roles and genetic potentials. The next step will be to characterise binned genomes by elucidating their actual transcriptional activity. In this aspect, metatranscriptome analyses will enable identification of predominantly transcribed genes which are believed to encode important functions within the biogas production process with respect to prevailing fermentation conditions. Accordingly, integrative analyses of deeply sequenced metagenome and metatranscriptome data will provide cultivation-independent insights into the performance of so far uncharacterized biogas community members.

## Additional files

10.1186/s13068-016-0565-3 Final operational taxonomic unit (OTU) table in BIOM format, format specification 1.0.0. Contained are clustered OTUs with specific OTU IDs, their supporting 16S rRNA gene sequence counts per sample, and as metadata taxonomic classification per OTU, descriptive labels per sample.
10.1186/s13068-016-0565-3 Phylogenetic tree of all available type strains with operational taxonomic units (OTUs) of *Thermotogae*, *Fusobacteria*, *Spirochaetes* and *Cloacimonetes* taxa of the studied biogas plants and their closest non-type strain relatives embedded. Type strains are in *black*, OTUs are in *red*, their closest relatives in *purple*, their closest sequenced relatives in *blue* and their closest type strain relatives in *green*. 16S rRNA gene sequence tree construction was done using the ARB [31] software.
10.1186/s13068-016-0565-3 ‘Alanine, Aspartate and Glutamate Metabolism’ with proteins encoded in the *Fusobacteria* bin marked in *green* and *red*. *Violet* marked proteins are uncertain. The analysis was done with the KEGG-based protein mapping tool implemented within the annotation system GenDB 2.0 [41].
10.1186/s13068-016-0565-3 Genes encoding proteins involved in energy generation found in the reference genome of *Defluviitoga tunisiensis* L3 [90] and their presence or absence within the *Thermotogae* genome bin.
10.1186/s13068-016-0565-3 Genes encoding proteins involved in life in the presence of oxygen, anaerobic lifestyle and energy generation found in the reference genome of *Candidatus Cloacamonas acidaminovorans* [89] and presence or absence of these within the *Cloacimonetes* genome bin.

## Abbreviations

BGP: biogas plant
CSTR: continuously stirred tank reactor
JGI: U.S. Department of Energy Joint Genome Institute
HAC-eq: acetic acid equivalents
KEGG: Kyoto encyclopaedia of genes and genomes
oDM: organic dry matter
OTU: operational taxonomic unit
QC: quality control
TIC: total inorganic carbon
VOA: volatile organic acids
