# Supplementary figures and images for: Identification and genome reconstruction of abundant distinct taxa in microbiomes from one thermophilic and three mesophilic production-scale biogas plants
Source: Biotechnol Biofuels. 2016 Jul 26;9:156. doi: 10.1186/s13068-016-0565-3 (PMC4960831; doi:10.1186/s13068-016-0565-3)

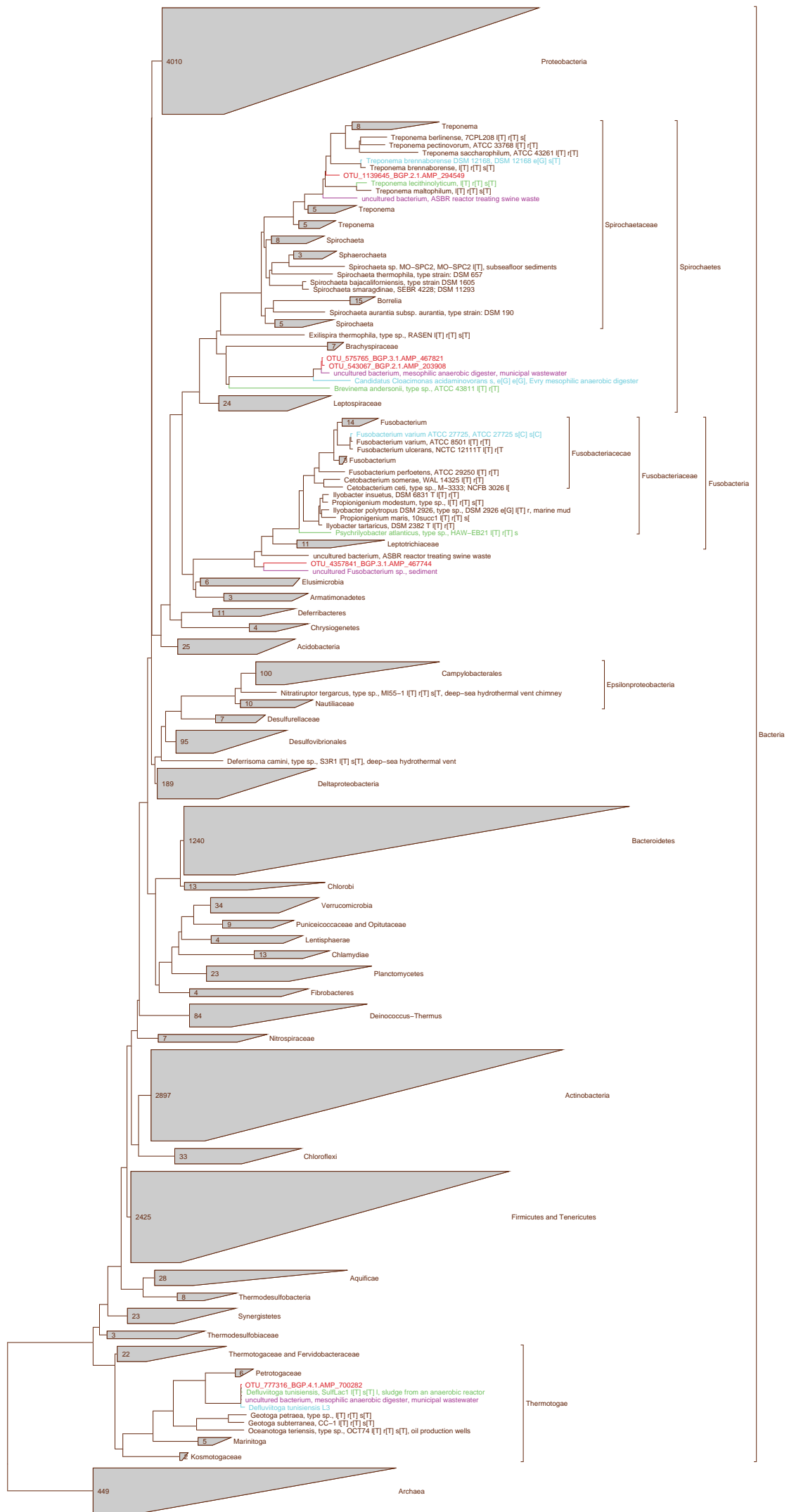

Supplement: Supplementary file 2 — 10.1186/s13068-016-0565-3 Phylogenetic tree of all available type strains with operational taxonomic units (OTUs) of Thermotogae, Fusobacteria, Spirochaetes and Cloacimonetes taxa of the studied biogas plants and their closest non-type strain relatives embedded. Type strains are in black, OTUs are in red, their closest relatives in purple, their closest sequenced relatives in blue and their closest type strain relatives in green. 16S rRNA gene sequence tree construction was done using the ARB [31] software. [file 13068_2016_565_MOESM2_ESM.pdf]

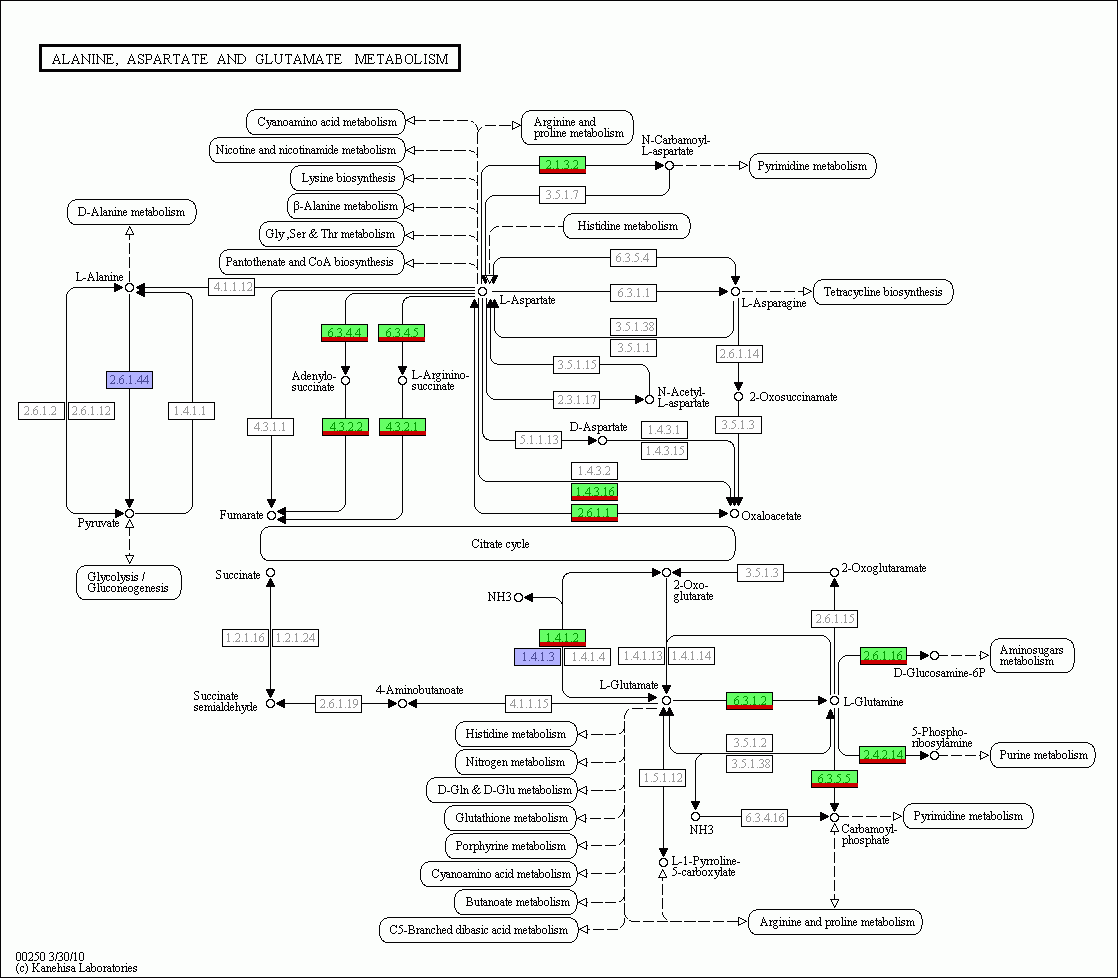

Supplement: Supplementary file 3 — 10.1186/s13068-016-0565-3 ‘Alanine, Aspartate and Glutamate Metabolism’ with proteins encoded in the Fusobacteria bin marked in green and red. Violet marked proteins are uncertain. The analysis was done with the KEGG-based protein mapping tool implemented within the annotation system GenDB 2.0 [41]. [file 13068_2016_565_MOESM3_ESM.png]
