# Supplementary material for: Identification and genome reconstruction of abundant distinct taxa in microbiomes from one thermophilic and three mesophilic production-scale biogas plants
Source: Biotechnol Biofuels. 2016 Jul 26;9:156. doi: 10.1186/s13068-016-0565-3 (PMC4960831; doi:10.1186/s13068-016-0565-3)
Supplement: Supplementary file 4 — 10.1186/s13068-016-0565-3 Genes encoding proteins involved in energy generation found in the reference genome of Defluviitoga tunisiensis L3 [90] and their presence or absence within the Thermotogae genome bin. [file 13068_2016_565_MOESM4_ESM.pdf]

Table S1: Genes encoding proteins involved in energy generation found in the reference genome of *Defluviitoga tunisiensis* L3 [Maus et al., 2016] and presence or absence of these within the *Thermotogae* genome bin.

Gene name	(Putative) gene product	*Thermotogae* bin
**Glucose fermentation**		
*xyl*EFK[1]	glucose/xylose transporter	Present
glk	glucokinase (EC: 2.7.1.2)	Present
pgi	phosphoglucose isomerase (EC: 5.3.1.9)	Present
pfk	Phosphofructokinase (EC: 2.7.1.11)	Present
fba	fructose bisphosphate aldolase (EC: 4.2.1.13)	Present
tpi	triosephosphate isomerase (EC: 5.3.1.1)	Absent
gap	glyceraldehyde 3-phosphate dehydrogenase (EC: 1.2.1.12)	Present
pgk	phosphoglycerate kinase (EC: 2.7.2.3)	Present
pgm	phosphoglucomutase (EC: 5.4.2.1)	Present
eno	enolase (EC: 4.2.1.11)	Present
pyk	pyruvate kinase (EC: 2.7.1.40)	Present
**Hexoses fermentation**		
tkt	transketolase (EC: 2.2.1.1)	Absent
rpi	Ribose 5-phosphate isomerase (EC: 5.3.1.6)	Absent
rpe	ribulose-phosphate 3-epimerase (EC: 5.1.3.1)	Present
*mel*A[2]	alpha galactosidase (EC: 3.2.1.22)	Absent
*scr*K	fructokinase (EC: 2.7.1.4)	Absent
**Lactose fermentation**		
*mal*EFG[3]	maltose/oligosaccharide transporter	Present
*lac*A	beta-galactosidase (EC: 3.2.1.23)	Present
*gal*K	galactokinase (EC: 2.7.1.6)	Present
*gal*T	galactose-1-phosphate uridylyltransferase (EC: 2.7.7.12)	Present
*gal*E	UDP-glucose 4-epimerase (EC: 5.1.3.2)	Present
**Galactan fermentation**		
*gan*A	arabinogalactan endo-1,4-beta-galactosidase (EC: 3.2.1.89)	Absent
**Stachyose fermentation**		
*mel*Z	alpha-glucosidase (EC: 3.2.1.20)	Absent
**Glycogen/Pullulan fermentation**		
*pul*A	pullanase (EC: 3.2.1.41)	Present
*amy*A	alpha amylase (extracellular) (EC: 3.2.1.1)	Present
*amy*B	alpha amylase (cytoplasmic) (EC: 3.2.1.1)	Present

**Mannosides fermentation**		
*mtp*EFGKL	Mannosides transporter	Present
*man*C	glycosylase (GH32)	Present
*man*A*oppABCDE*	mannose-6-phosphate isomerase (EC: 5.3.1.8)	Present
**Trehalose fermentation**		
*tre*S	trehalose synthase (EC: 5.4.99.16)	Absent
**Lichenan fermentation**		
dpp/oppABCDE[4]	lichenan transporter	Absent
*lic*B	licheninase (EC: 3.2.1.73)	Absent
*bgl*B	thermostable beta-glucosidase (EC: 3.2.1.21)	Absent
**Cellulose fermentation**		
*dpp/opp*ABCDF[4]	cellobiose transporter	Absent
*cel*5A	endo-1,4-beta-D-glucanase (EC: 3.2.1.4)	Absent
*cbh*1	cellulose 1,4-beta-cellobiosidase (EC: 3.2.1.91)	Absent
*bgl*A	cytosolic beta-glucosidase (EC: 3.2.1.21)	Absent
**Chitin fermentation**		
*chi*EFG	chitinobiose transporter	Absent
*chi*A	chitinase (EC: 3.2.1.14)	Absent
*nag*Z	beta-N-acetylhexosaminidase (EC: 3.2.1.52)	Absent
*nag*A	N-acetylglucosamine-6-phosphate deacetylase (EC: 3.5.1.25)	Absent
*nag*B	glucosamine-fructose-6-phosphate aminotransferase (EC: 2.6.1.16)	Absent
**Oligo galacturonates fermentation**		
*agu*EFG	oligo galacturonates transporter	Present
*agu*A	alpha-glycosidase (EC: 3.2.1.20)	Absent
*uxa*C	glucuronate isomerase (EC: 5.3.1.19)	Absent
*uxu*B	D-mannonate oxidoreductase (EC: 1.1.1.57)	Present
*uxu*A	mannonate dehydratase (EC: 4.2.1.8)	Absent
*kdg*K	2-keto-3-deoxygluconate kinase (EC: 2.7.1.178)	Present
**Xylan fermentation**		
*xtp*ELKGF	xylan oligosaccharides transporter	Absent
*xlo*ELKGF	xylan oligosaccharides transporter	Absent
*xyn*A	beta-xylosidase (EC: 3.2.1.8)	Present
*bxl*B	beta-xylosidase (EC: 3.2.1.37)	Absent
**Xylose fermentation**		
*xyl*A	xylose isomerase (EC: 5.3.1.5)	Present
*xyl*B	xylulokinase (EC: 2.7.1.17)	Present
**Arabinosides fermentation**		

*ara*EFG	arabinosides transporter	Present
*abf*A	alpha-L-arabinofuranosidase (EC: 3.2.1.55)	Present
*ara*A	L-arabinose isomerase (EC: 5.3.1.4)	Absent
*ara*B	ribulokinase (EC: 2.7.1.16)	Present
*ara*D	L-ribulose-5-phosphate 4-epimerase (EC: 5.1.3.4)	Present
*ara*M	aldose 1-epimerase (EC: 5.1.3.3)	Present
**Pyruvate metabolism**		
*por*ABCD[5]	pyruvate ferredoxin oxidoreductase (EC: 1.2.7.1)	Present
ack	acetate/propionate kinase (EC: 2.7.2.15)	Present
ldh	lactate dehydrogenase (EC: 1.1.1.27)	Present
adh	alcohol dehydrogenase (EC: 1.1.1.2)	Present
**Incomplete TCA cycle**		
idh	isocitrate dehydrogenase (EC: 1.1.1.41)	Present
*kor*AB	2-oxoglutarate oxidoreductase (EC: 1.2.7.3)	Present
*fum*A	fumarate hydratase (EC: 4.2.1.2)	Present
**Hydrogen production**		
*hyd*ABG	[Fe-Fe] hydrogenase (EC: 1.12.1.4)	Present
**Propionate metabolism**		
mcm	methylmalonyl-CoA mutase (EC: 5.4.99.2)	Present
mmc	methylmalonyl-coA carboxytransferase (EC: 2.1.3.1)	Present
*mmc*E	methylmalonyl-coA epimerase (EC: 5.1.99.1)	Present
*pdu*L	phosphate propanoyltransferase (EC: 2.3.1.222)	Present
*tdc*D	propionate kinase/acetate kinase (EC: 2.7.2.15)	Present
**Miscellaneous**		
*rbs*K	ribokinase (EC: 2.7.1.15)	Present
zwf	glucose-6-phosphate 1-dehydrogenase (EC: 1.1.1.49)	Present
pgl	6-phosphogluconolactonase (EC: 3.1.1.31)	Absent
gnd	6-phosphogluconate dehydrogenase (EC: 1.1.1.44)	Present
*glm*M	phosphoglucosamine mutase (EC: 5.4.2.10)	Present
*glm*U[6]	N-acetylglucosamine-1-phosphate uridyltransferase/glucosamine-1-phosphate acetyltransferase (EC: 2.3.1.157)	Present

1 the transport system XylEFK was showed to import glucose and xylose.

2 the gene *mel*A was predicted to encode multifunctional alpha galactosidase (3.2.1.22).

3 the substrate binding protein MalE of the transport system MalEFG was described to bind different carbohydrates. Therefore, differed sugars are predicted to be imported *via* the MalEFG transport system.

4 predicted genes encoding not further characterized Dpp/Opp family transport system proposed to be responsible for the import of the several carbohydrates including β - glucosides and cellobiose.

5 the gene encoding the PorABCD enzyme was shown to possess a dual function.

6 the gene *glm*U was predicted to encode bifunctional enzyme.
