# Supplementary material for: Identification and genome reconstruction of abundant distinct taxa in microbiomes from one thermophilic and three mesophilic production-scale biogas plants
Source: Biotechnol Biofuels. 2016 Jul 26;9:156. doi: 10.1186/s13068-016-0565-3 (PMC4960831; doi:10.1186/s13068-016-0565-3)
Supplement: Supplementary file 5 — 10.1186/s13068-016-0565-3 Genes encoding proteins involved in life in the presence of oxygen, anaerobic lifestyle and energy generation found in the reference genome of Candidatus Cloacamonas acidaminovorans [89] and presence or absence of these within the Cloacimonetes genome bin. [file 13068_2016_565_MOESM5_ESM.pdf]

Table S2: Genes encoding proteins involved in Life in the presence of oxygen, Anaerobic lifestyle and energy generation found in the reference genome of *Candidatus* Cloacamonas acidaminovorans [Pelletier et al. (2008)] and presence or absence of these within the *Cloacimonetes* genome bin.

Gene name	Encoded protein	*Cloacimonetes* bin
**Life in the presence of oxygen**		
dfx	Superoxide reductase (EC: 1.15.1.2)	Present
rbr	Ruberythrin (EC: 1.11.1.1)	Present
rub	Rubredoxin	Present
ahpC	Peroxiredoxin (EC: 1.11.1.15)	Present
trx	(thiol reductase) Thioredoxin	Present
trxB	Thioredoxin reductase (EC: 1.8.1.9)	Present
fprA	Rubredoxin-oxygen oxidoreductase (EC: 1.15.1.2)	Present
Cloam0913[1]	Class II ribonucleotide reductase (putative)	Present
**Anaerobic lifestyle**		
nrdD	Class III ribonucleotide reductase (EC: 1.17.4.2)	Present
nrdG	Class III ribonucleotide reductase-activating enzyme (EC: 1.97.1.-)	Present
por	Pyruvate ferredoxin oxidoreductase (EC: 1.2.7.1)	Present
korC	2-Ketoglutarate ferredoxin oxidoreductase γ-subunit (EC: 1.2.7.3)	Present
korB	2-Ketoglutarate ferredoxin oxidoreductase β-subunit (EC: 1.2.7.3)	Present
korA	2-Ketoglutarate ferredoxin oxidoreductase α-subunit (EC: 1.2.7.3)	Present
korD	2-Ketoglutarate ferredoxin oxidoreductase δ-subunit (EC: 1.2.7.3)	Present
aorB	Aldehyde ferredoxin oxidoreductase, tungsten containing (EC: 1.2.7.5)	Present
aorA	Aldehyde ferredoxin oxidoreductase, tungsten containing (EC: 1.2.99.6)	Present
vorD	Branched-chain ketoacid ferredoxin oxidoreductase δ-subunit (EC: 1.2.99.5)	Present

vorC	Branched-chain ketoacid ferredoxin oxidoreductase γ-subunit (EC: 1.2.99.5)	Present
vorB	Branched-chain ketoacid ferredoxin oxidoreductase β-subunit (EC: 1.2.99.5)	Present
vorA	Branched-chain ketoacid ferredoxin oxidoreductase α-subunit (EC: 1.2.99.5)	Present
iorB	Indolepyruvate ferredoxin oxidoreductase (EC: 1.2.7.8)	Present
iorA	Indolepyruvate ferredoxin oxidoreductase (EC: 1.2.7.8)	Present
**Energy**		
hymC	Fe-only hydrogenase α-subunit (EC: 1.12.7.2)	Present
hymB	Fe-only hydrogenase β-subunit (EC: 1.12.7.2)	Present
hymA	Fe-only hydrogenase γ-subunit (EC: 1.12.7.2)	Present
hydEF	Fe-only hydrogenase, assembly protein	Present
hydG	Fe-only hydrogenase, assembly protein	Present
Cloam1768[1]	Methylmalonyl-CoA-decarboxylase (putative) α-subunit (EC: 4.1.1.41)	Present
Cloam1770[1]	Methylmalonyl-CoA-decarboxylase (putative) γ-subunit (EC: 4.1.1.41)	Present
Cloam1771[1]	Methylmalonyl-CoA-decarboxylase (putative) β-subunit (EC: 4.1.1.41)	Present
Cloam1052[1]	ATP synthase, subunit A1 (EC: 3.6.3.15)	Present
Cloam1053[1]	ATP synthase, subunit B1 (EC: 3.6.3.15)	Present
Cloam1054[1]	ATP synthase, subunit D	Present
Cloam1055[1]	ATP synthase, subunit I, putative (EC: 3.6.3.14)	Present
Cloam1056[1]	ATP synthase, subunit K, putative	Present
hppA	Pyrophosphate-energised proton pump (EC: 3.6.1.1)	Present

[1] Name of genetic determinant in *Candidatus* Cloacamonas acidaminovorans genome, no other name given.
